# Rapid Change in Mental Status in a Patient with Hypereosinophilia

**DOI:** 10.1155/2017/6936709

**Published:** 2017-01-10

**Authors:** Hanyin Wang, John K. Erban

**Affiliations:** ^1^Department of Internal Medicine, Tufts Medical Center, 800 Washington St., Boston, MA 02111, USA; ^2^Division of Hematology/Oncology, Tufts Medical Center, 800 Washington St., Boston, MA 02111, USA

## Abstract

We present the case of a 48-year-old female with acute onset altered mental status, who was found to have eosinophilia, elevated troponin, and embolic strokes. Extensive testing for autoimmune, infectious, and coronary artery etiologies was unremarkable. After a cardiac MRI revealed focal myocardial hyperenhancement, the patient underwent an endomyocardial biopsy with findings consistent with eosinophilic myocarditis. The patient was diagnosed of idiopathic hypereosinophilic syndrome and started on prednisone and apixaban. Our case highlights the importance of considering hypereosinophilic syndrome when eosinophilia is associated with multisystem impairments, as tissue biopsy is usually required to diagnose this rare condition.

## 1. Introduction

Eosinophilia is defined as an increase in the peripheral absolute eosinophil count to greater than 0.5 × 10^9^/L. A number of clinical conditions can lead to eosinophilia, including drug hypersensitivity, allergic disorders, infection (particularly parasitic helminth infection), neoplastic disorders, and immune dysregulations [1, 2]. Increased numbers of eosinophils can infiltrate tissues, release cellular mediators, and cause organ damage and dysfunction [1].

The hypereosinophilic syndrome (HES) is a group of conditions characterized by overproduction of eosinophils and evidence of end organ manifestations attributable to the eosinophilia. HES is a rare disease with estimated prevalence between 0.315 and 6.3 per 100,000 [3]. Due to its variable presentation with potential to involve multiple systems including skin, heart, lungs, gastrointestinal tract, and central and peripheral nervous systems [4], HES remains to be a diagnostic challenge.

We present a case of a 48-year old female hospitalized with acute change in mental status, found to have eosinophilia, embolic strokes, and elevated troponin. After extensive evaluation including endomyocardial biopsy, the patient was diagnosed of idiopathic HES.

## 2. Case Presentation

A 48-year-old female with past medical history significant for psoriasis was brought in by ambulance to the emergency room with 1 day of acute change in mental status. She was found to be walking naked in the room, incontinent of urine and stool, and answering questions bizarrely. She was not on any home medication, but, 3 weeks prior to current presentation, she received minocycline for acne breakout. She traveled to Caribbean area one year ago. On presentation to emergency room, she was afebrile. Neurological exam was nonfocal; however, she only scored 18/30 on Montreal cognitive assessment (normal is ≧ 26), with deficits in memory and executive skills.

Blood investigations revealed white blood cell count 11.3 × 10^9^/L, eosinophil count 3.2 × 10^9^/L, Troponin I 1.78 *μ*g/L, and NT-ProBNP 6387 ng/L. Creatinine, liver enzymes, C-reactive protein, and Vitamin B12 level were normal. Peripheral blood smear was unrevealing. EKG showed sinus rhythm and inverted T waves in anterolateral leads. Head CT initially showed no acute process, but a subsequent head MRI demonstrated multiple small acute infarcts in bilateral frontal, parietal, occipital, and temporal lobes, likely embolic in etiology.

She was started on low molecular weight heparin, aspirin, and atorvastatin for presumed acute coronary syndrome. Further testing for antineutrophil cytoplasmic antibodies, antinuclear antibodies, cardiolipin antibodies, and stool ova and parasites was negative, as well as serologies for Strongyloides, Trichinella, Schistosomiasis, HLTV, and HIV. Transesophageal echocardiogram demonstrated increased left ventricular trabeculation, normal ejection fraction, and no interatrial shunt or intracardiac thrombus. A lumbar puncture was performed and cerebrospinal fluid analysis was unremarkable with negative PCR results for herpes simplex virus and varicella-zoster virus. Left heart catheterization was performed which showed clean coronary arteries. At this point, ivermectin was started for possible Strongyloides infection and the patient was transferred to a tertiary hospital with cardiac MRI capacity.

**Figure 1:**
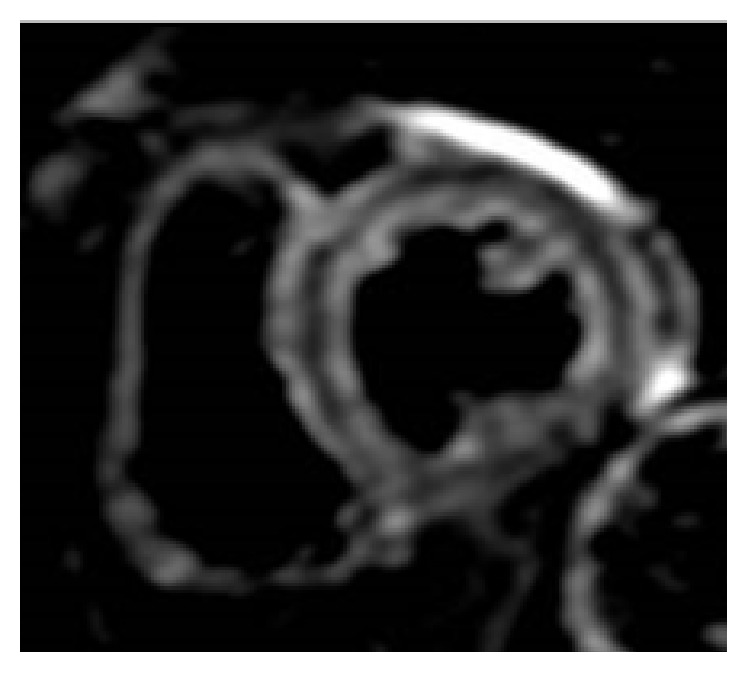
T2-weighted sequences cardiac MRI of the patient. There is a bright white rim of enhancement lining the lateral wall of left ventricle, which is consistent with subendocardial edema.

A gadolinium-enhanced cardiac MRI revealed circumferential subendocardial edema from the mid cavity to apex, most prominently in the lateral wall (Figure 1). The patient underwent endomyocardial biopsy, and pathology was compatible with eosinophilic myocarditis. Hematology team recommended testing for blood platelet-derived growth factor receptor alpha (PDGFRA) and BCR/ABL1 which were negative and flow cytometry which showed a normal population of cells only. The patient was diagnosed of idiopathic HES. She was started on prednisone 60 mg Qday with plan of slow taper as well as apixaban for anticoagulation. At the time of discharge, her mental status largely improved and eosinophil count had returned to normal range. Repeat cardiac MRI 3 months later showed resolution of prior subendocardial edema, consistent with recovery of myocarditis.

## 3. Discussion

Since Chusid et al. established the first formal diagnostic criteria for HES in 1975 [5], the definition of HES has evolved over the past 40 years [6]. In The Year 2011 Working Conference on Eosinophil Disorders and Syndromes, hypereosinophilia (HE) is defined as an absolute eosinophil count greater than 1.5 × 10^9^/L on two examinations (interval > 1 month) and/or pathologic confirmation of tissue HE [7]. The HESs are defined as conditions with HE and eosinophil-mediated organ damage and/or dysfunction, provided that other alternative causes for organ damage have been ruled out [7]. HES can be classified into several clinical variants including myeloproliferative HES, lymphocytic HES, overlap HES, associated HES, familial HES, and idiopathic HES [6, 8].

Neurologic involvement in HES patients is highly variable. Interestingly, there are two components of central nervous systems dysfunction in our case: encephalopathy as evidenced by global confusion and embolic strokes as demonstrated by head MRI. Both patterns have been reported in patients with HES. Encephalopathy usually occurs early in the course of syndrome, and embolic strokes appear to be parallel with the cardiac involvement [9]. Cardiac pathology of HES has been divided into three stages: acute necrosis, thrombosis, and fibrosis [10]. In the thrombosis stage, thrombi form along the damaged endocardium and can cause strokes and other embolic events [11]. Overall, approximately 25% of patients with HES will develop thromboembolic complications that 5% to 10% died of them [10].

Minocycline is a semisynthetic tetracycline and is known to cause eosinophilia. One important aspect in our case is to differentiate between HES and minocycline-induced hypersensitivity syndrome. While eosinophilic pneumonia and hepatitis are relatively common in minocycline-induced adverse reactions, to date, there are only four case reports of minocycline-induced myocarditis, and this condition is usually not associated with thromboembolic complications [12–15].

Endomyocardial biopsy remains the gold standard to diagnose cardiac involvement in HES [11]. However, it is well-recognized that biopsies for HES may be low-yield, because of either sampling error (e.g., primary involvement of left ventricle rather than right ventricle) or extensive eosinophil degranulation [16]. In recent years, cardiac MRI has emerged as a powerful noninvasive modality to diagnose eosinophilic myocarditis. Gadolinium-enhanced cardiac MRI is capable of detecting myocardial fibrosis and inflammation and is also more sensitive in detecting ventricular thrombi than echocardiography [17–20]. There have been case reports where the diagnosis of HES is made with gadolinium-enhanced MRI alone in the appropriate clinical setting, thus obviating the need for performing endomyocardial biopsy [18, 20]. In our case, given a high index of suspicion based on the patient's HE, multiorgan involvement, thromboembolic complication, and characteristic cardiac MRI findings, it probably would be reasonable to initiate treatment for HES without pursing endomyocardial biopsy.

Treatment of HES should be guided by clinical variant. While systemic steroids remain the first-line therapy for most forms of HES, efforts to treat underlying causes (e.g., parasite helminth infection, sarcoidosis, and IgG-4 related disease) should be made for secondary HES [8]. Identification of patients with PDGFRA-positive myeloproliferative neoplasms is important, since Imatinib would be the first-line therapy for these patients [8]. Second-line options for steroid-resistant HES include hydroxyurea, interferon-*α*, and novel immunomodulatory agents [8, 21]. When life-threatening manifestations are present or imminent, steroid therapy should be started immediately without waiting the 1-month interval to confirm blood HE [8]. For patients with a history of potential exposure to Strongyloides as in our case, empiric treatment with ivermectin for 2 days is reasonable to prevent steroid-associated hyperinfection [16]. Anticoagulation should be started for patients who have or are at risk of thromboembolic complication, and duration of anticoagulation should be determined by the activity of endomyocardial disease [10].

Our case highlights the need to consider HES as one of the potential causes of eosinophilia associated with multisystem impairments. There is inherent difficulty in diagnosing this rare condition with variable clinical presentations, and tissue biopsy is usually necessary to make the diagnosis. Our patient underwent multiple imaging studies, left heart catheterization, transfer to a tertiary hospital, and eventually endomyocardial biopsy before a diagnosis of HES could be made. With advance in molecular characterization of HES and development of novel immunomodulatory agents, correct identification of clinical variant is critical to guide treatment for HES.
